# A change in brain white matter after shunt surgery in idiopathic normal pressure hydrocephalus: a tract-based spatial statistics study

**DOI:** 10.1186/s12987-016-0048-8

**Published:** 2017-01-30

**Authors:** Shigenori Kanno, Makoto Saito, Tomohito Kashinoura, Yoshiyuki Nishio, Osamu Iizuka, Hirokazu Kikuchi, Masahito Takagi, Masaki Iwasaki, Shoki Takahashi, Etsuro Mori

**Affiliations:** 1 Department of Neurology, Southmiyagi Medical Center, 38-1, Aza-nishi, Shibata, Miyagi 989-1253 Japan; 2 Department of Behavioural Neurology and Cognitive Neuroscience, Tohoku University Graduate School of Medicine, Sendai, Japan; 3 Department of Neurosurgery, Tohoku University Graduate School of Medicine, Sendai, Japan; 4 Department of Diagnostic Radiology, Tohoku University Graduate School of Medicine, Sendai, Japan

**Keywords:** Idiopathic normal pressure hydrocephalus, Diffusion tensor imaging, White matter, Ventriculoperitoneal shunt, Lumboperitoneal shunt

## Abstract

**Background:**

The aim of this study was to elucidate changes in cerebral white matter after shunt surgery in idiopathic normal pressure hydrocephalus (INPH) using diffusion tensor imaging (DTI).

**Methods:**

Twenty-eight consecutive INPH patients whose symptoms were followed for 1 year after shunt placement and 10 healthy control (HC) subjects were enrolled. Twenty of the initial 28 INPH patients were shunt-responsive (SR) and the other 8 patients were non-responsive (SNR). The cerebral white matter integrity was detected by assessing fractional anisotropy (FA) and mean diffusivity (MD). The mean hemispheric DTI indices and the ventricular sizes were calculated, and a map of these DTI indices was created for each subject. The DTI maps were analysed to compare preshunt INPH with HC and preshunt INPH with 1 year after shunt placement in each INPH group, using tract-based spatial statistics. We restricted analyses to the left hemisphere because of shunt valve artefacts.

**Results:**

The ventricles became significantly smaller after shunt placement both in the SR and SNR groups. In addition, there was a significant interaction between clinical improvement after shunt and decrease in ventricular size. Although the hemispheric DTI indices were not significantly changed after shunt placement, there was a significant interaction between clinical improvement and increase in hemispheric MD. Compared with the HC group, FA in the corpus callosum and in the subcortical white matter of the convexity and the occipital cortex was significantly lower in SR at baseline, whereas MD in the periventricular and peri-Sylvian white matter was significantly higher in the SR group. Compared with the pre-operative images, the post-operative FA was only decreased in the corona radiata and only in the SR group. There were no significant regions in which DTI indices were altered after shunt placement in the SNR group.

**Conclusions:**

Brain white matter regions in which FA was decreased after shunt placement were in the corona radiata between the lateral ventricles and the Sylvian fissures. This finding was observed only in shunt-responsive INPH patients and might reflect the plasticity of the brain for mechanical pressure changes from the cerebrospinal fluid system.

**Electronic supplementary material:**

The online version of this article (doi:10.1186/s12987-016-0048-8) contains supplementary material, which is available to authorized users.

## Background

Neuropathological findings in idiopathic normal pressure hydrocephalus (INPH) are generally consistent with white matter damage, regardless of the underlying, yet unknown, pathophysiological mechanisms involved in INPH [1–5]. Diffusion tensor imaging (DTI) has recently been applied to evaluate white matter damage in INPH because DTI is a useful MRI technique that can reflect the structural integrity and interstitial space of the white matter by detecting the directionality of extracellular water diffusion [fractional anisotropy (FA)] and of free water diffusion [mean diffusivity (MD)] [6–9]. However, it has been found that these DTI indices could not only reflect the microstructural damage of fibres, such as axonal degeneration or ischaemic demyelination, which is generally represented by a low FA value, because several recent DTI studies reported that FA values of the periventricular corticospinal tract (CST) in INPH patients were higher compared to those in healthy controls [10–12]. Hattigen et al. postulated that mechanical compaction of the CST due to ventricular dilatation might increase fibre density and directionality of diffusion [10]. Hattori et al. noted that the decreased integrity of the superior longitudinal fasciculus, which crosses the CST at a level of the corona radiata, might increase the FA value of the CST [11]. Therefore, interpretations of FA value abnormalities in INPH patients should be affected by the different effects of mechanical pressure depending on the orientation and organization of the fibre bundles. Investigation of regional changes of DTI indices after shunt placement is needed.

Another issue among DTI studies in INPH is that most of the studies were limited by inhomogeneity in aetiology and differences in DTI analysis methods [9–13]. In the DTI studies for INPH, anatomical region of interest (ROI) approaches have been used because of the severe morphological changes that occur in the brain. However, the brain regions selected for analyses in these studies were often arbitrary and inconsistent with each other. On the other hand, although at present there is no established method for achieving accurate registration of images in INPH patients, tract-based spatial statistics (TBSS) [14], which was developed for analysing FA images from multiple subjects with INPH, have been used for voxel-based DTI analysis. The TBSS has a function that can reverse each voxel in normalized space back to its original location in native space (back-projection). This function is useful for confirming accurate registration of images in which severe deformations occur, such as in INPH patients [15]. In the present study, we attempted to elucidate a change in brain white matter involvement in INPH patients by shunt placement using DTI. We also investigated the difference between shunt responders and non-responders by applying TBSS including back-projection for tract-based DTI and mean hemispheric DTI analyses [9].

## Methods

### Subjects

Fifty (20 female/30 male) consecutive patients with INPH who underwent shunt surgery at Tohoku University Hospital between August 2006 and October 2010 and 10 healthy control (HC) subjects (mean age: 65.1 ± 3.6 years, 5 females and 5 males) were enrolled in this study. The patients were diagnosed with probable INPH by board-certified neurologists based on the diagnostic criteria established according to the Japanese Clinical Guidelines for INPH [16]. The criteria for probable INPH are as follows: (1) >60 years of age; (2) gait disturbance, dementia, and/or urinary incontinence; (3) ventricular dilatation (Evans index >0.3) with a narrow cerebrospinal fluid (CSF) space in the superior convexity; (4) CSF pressure <200 mm H_2_O with normal CSF cell counts and protein levels; (5) the absence of other diseases that may account for such symptoms; (6) the lack of a previous history of illness that may cause ventricular dilatation; and (7) a positive CSF tap test.

Shunt implantation was conducted using a Codman-Hakim programmable valve with a Siphon-Guard (Codman and Shurtleff, Johnson and Johnson Inc.). A shunt tube was inserted in the right anterior horn of the lateral ventricle in all the patients who underwent a ventriculoperitoneal (VP) shunt procedure and in the lumbar subarachnoid space in all the patients who underwent a lumboperitoneal (LP) shunt procedure. Post-operatively, the patients were followed at the outpatient clinic, and the pressure setting of their programmable value was adjusted in a stepwise manner. Pressure adjustments were made repeatedly until the optimal pressure for each patient was attained. Thirty-five (18 female/17 male) of the initial 50 INPH patients were re-evaluated approximately 1 year after shunt surgery. The other 15 patients withdrew because of refusal, heart attack, cancer, or residential relocation. Twenty-seven (10 female/17 male) of the re-evaluated INPH patients showed significant shunt responsiveness, which is defined as an improvement by one or more points on the total score of the idiopathic Normal pressure hydrocephalus grading scale (iNPHGS) [17] at 1 year after shunt placement. However, we excluded 7 patients with cerebral vascular lesions, either focal or multiple lesions, both with abnormal intensity as observed by fluid attenuated inversion recovery (FLAIR) images and with hypointensity as observed by T1-weighted images, to avoid the possibility that the DTI parameters and clinical symptoms would be affected by a vascular brain disease. However, we did not exclude the patients only with periventricular hyperintensity as observed by FLAIR images. Consequently, the improving 20 patients (shunt responders: SR) and not improving 8 patients (shunt non-responders: SNR) with INPH after shunt placement were included in this study. The demographic characteristics of the patients with INPH at baseline are shown in Table 1.

**Table 1:** Demographics of patients at baseline

Variables	INPH (n = 20)	INPH (n = 8)	df	p value^a^
	Responder (SR)	Non-responder (SNR)		
Age in years, mean (SD)	75.5 (5.2)	76.6 (3.9)	26	NS
Sex, female/male	8/12	1/7	1	0.023
Education years, mean (SD)	10.4 (2.9)	8.8 (3.2)	26	NS
CSF shunt placement (VP/LP)	13/7	6/2	1	NS
TUG, mean (SD)				
Time to complete (s)	14.7 (4.1)	15.6 (3.1)	26	NS
Number of steps	21.9 (7.0)	22.6 (4.0)	26	NS
iNPHGS, median (range)				
Gait disturbance	2.0 (2–3)	2.0 (1–4)		NS
Cognitive disturbance	3.0 (0–3)	2.5 (1–3)		NS
Urinary disturbance	1.5 (0–3)	2.0 (0–4)		NS
Total	6.5 (2–9)	7.0 (4–9)		NS
MMSE, mean (SD)	21.5 (4.6)	22.3 (5.1)	26	NS
FAB, mean (SD)	10.7 (2.3)	12.9 (3.1)	26	NS
Left VV/ICV ratio (%), mean (SD)	8.29 (1.99)	7.19 (1.64)	26	NS
Left hemispheric FA, mean (SD)	0.386 (0.022)	0.380 (0.024)	26	NS
Left hemispheric MD (×10^−3^ mm^2^/s), mean (SD)	0.813 (0.036)	0.829 (0.068)	26	NS

*INPH* idiopathic normal pressure hydrocephalus, iNPHGS idiopathic normal pressure hydrocephalus grading scale, *VP* ventriculoperitoneal, *LP* lumboperitoneal, *TUG* Timed “Up and GO” test, *MMSE* Mini-Mental State Examination, *FAB* frontal assessment battery, *Left VV/ICV ratio* the ratio of the volume of the left lateral ventricle + the cerebral aqueduct + the left side region of the third and fourth ventricles and the volume of the left half intracranial space, *FA* fractional anisotropy, *MD* mean diffusivity, *SD* standard deviation, *df* degrees of freedom, *NS* not significant

^a^Paired Student’s t test was used except for iNPHGS (Mann–Whitney U test)

^b^Pearson’s Chi squared test was used for sex and CSF shunt placement ratios

### Clinical assessments

In the present study, clinical measures were assessed prior to performing both CSF removal and shunt placement and re-assessed approximately 1 year after shunt placement. In addition to the iNPHGS, we administered a gait test and a series of standard neuropsychological tests, including the Timed Up and Go (TUG) test [18], the Mini-Mental State Examination (MMSE) [19], and the frontal assessment battery (FAB) [20]. We used the FAB because it has been reported that executive dysfunction is a characteristic feature of the cognitive impairment in patients with INPH [21, 22].

### MRI procedure

Similar to the clinical assessment, cranial MRI scans were performed at baseline and approximately 1 year after shunt placement in the patients with INPH. Three-dimensional spoiled gradient echo (3D-SPGR) imaging and diffusion-weighted imaging (DWI) data were acquired via a single-shot spin echo-type echo planar imaging sequence with a GE Signa 1.5 Tesla MRI unit (General Electric Company, Milwaukee, WI, USA). The imaging parameters used for 3D-SPGR imaging were TR 20 ms, TE 4.1 ms, 1.5 mm thickness/0.0 sp by 108 slices and no intersection gap, FOV 21 × 21 cm, and matrix 256 × 256. The imaging parameters used for the acquisition of the DWI data were TR 15,000 ms, TE 52.8 ms, 2.5 mm thickness/0.0 sp by 50 slices and no insertion gap, FOV 23 × 23 cm, and matrix 256 × 256. The DWI data were acquired along 13 gradient directions with b = 1000 s/mm^2^. One volume was acquired with no diffusion weighting (b = 0 s/mm^2^). Two sets of the DWI (each set using same gradient directions) and no diffusion weighting data were acquired for subsequent averaging to improve the signal-to-noise ratio.

### MRI data processing

We restricted analyses to the left brain because the post-operative images of the right brain could be distorted by shunt valve artefacts except when comparing between the pre-operative DTI measures of the patients with INPH and those of the HC subjects. The left half volumes of the intracranial space and of the ventricles (the left lateral ventricle, the cerebral aqueduct, and the left side region of the third and fourth ventricles) that were obtained via 3D-SPGR imaging were measured manually using MRIcron [23] and Wacom™ tablets (Cintiq 12WX). The left volume of ventricles/intracranial volume (VV/ICV) ratio, which is calculated by dividing the left half volume of the ventricles (VV) by the left half volume of the intracranial space (ICV) and expressed as a percentage of ICV, was used as an index of the ventricular dilatation for each subject.

All diffusion images in each subject were aligned with the initial b0 image, and we used motion correction and registration software (eddy current correction) from the FSL software package [24]. The corrected images for each direction, which were acquired in two scans, were averaged to improve the signal-to-noise ratio. MD and FA maps were calculated from the mean diffusion weighted images for each direction using DTI calculation software from the FSL software package (DTIFIT Reconstruct diffusion tensors software). The mean MD and FA values of all of the left supratentorial white matter (the left hemispheric FA and MD), which was defined as the regions of the voxels with white matter probability values greater than 0.95 based on the SPM8 segmentation results of 3D-SPGR images (new segmentation software), were calculated from the co-registered MD and FA maps for each subject according to the method of Kanno et al. [9].

### TBSS analyses

Regional comparisons of the FA and MD maps between baseline and 1 year after shunt placement were performed using TBSS. The initial step of TBSS consisted of determining the most representative FA map (the most typical map of the subject in the analysed groups) as the one needing the least warping for all the other maps to align to it. This map was used as the target image, and the FA maps of all the patients and the HC subjects were nonlinearly transformed into the space of the target image. The transformed FA maps were averaged to create a mean FA skeleton of white matter tracts using an algorithm that found local FA maxima along the perpendicular direction of a white matter tract. An FA threshold of 0.2 was then used to differentiate between grey and white matter. Each patient’s warped FA map was projected onto the mean FA skeleton, and the final image was normalized to the Montreal Neurological Institute (MNI) space using FLIRT [25]. We used the TBSS results for the FA maps to analyse the MD maps of all the patients and the HC subjects.

### Statistical analyses and back-projection

Comparisons of the baseline MMSE scores, FAB scores, TUG completion times, TUG numbers of steps, left VV/ICV ratios, and left hemispheric FAs and MDs between the SR and SNR groups were performed using a two-tailed Student’s t test except for iNPHGS total and sub-scores (using the Mann–Whitney U test). For the baseline left VV/ICV ratio and left hemispheric DTI measures, comparisons were made between the SR or SNR groups, and the HC group. A two-tailed Student’s t test was used for comparison of the baseline and post-operative data between the patients following VP shunt surgery and those following LP shunt surgery in the SR group except for sex (using the Chi squared test) and iNPHGS total and sub-scores (using the Mann–Whitney U test).

A paired two-tailed Student’s *t* test was used for comparison between the baseline and post-operative data except for iNPHGS total and sub-scores (using the Wilcoxon signed rank test) in the SR and SNR groups. In addition, a repeated measure of analysis of variance (ANOVA) was used to analyse influences of shunt effectiveness on the change in ventricle size and hemispheric DTI measures. Statistical analyses were performed using JMP pro statistics software (version 11.00; SAS Institute Inc., Cary, NC, USA), and the statistical significance was defined for p values <0.05.

In the TBSS analyses, the baseline FA and MD maps of the SR and SNR groups were compared to those of the HC group using two sample t tests. In addition, comparisons of the baseline FA and MD maps between the SR and SNR groups were performed using two sample t tests. In these analyses, age and sex were included as nuisance variables [26, 27]. Furthermore, the FA and MD maps in the patients with INPH were compared between baseline and 1 year after shunt placement in each group using a paired t test. All the analyses were performed using the threshold-free cluster enhancement (TFCE) option of the randomize program in TBSS, which performs a permutation test developed for a general linear model. The number of permutations was set at 5000, and the significance threshold of the comparisons was set at p < 0.05 (family-wise error correction).

**Fig. 1:**
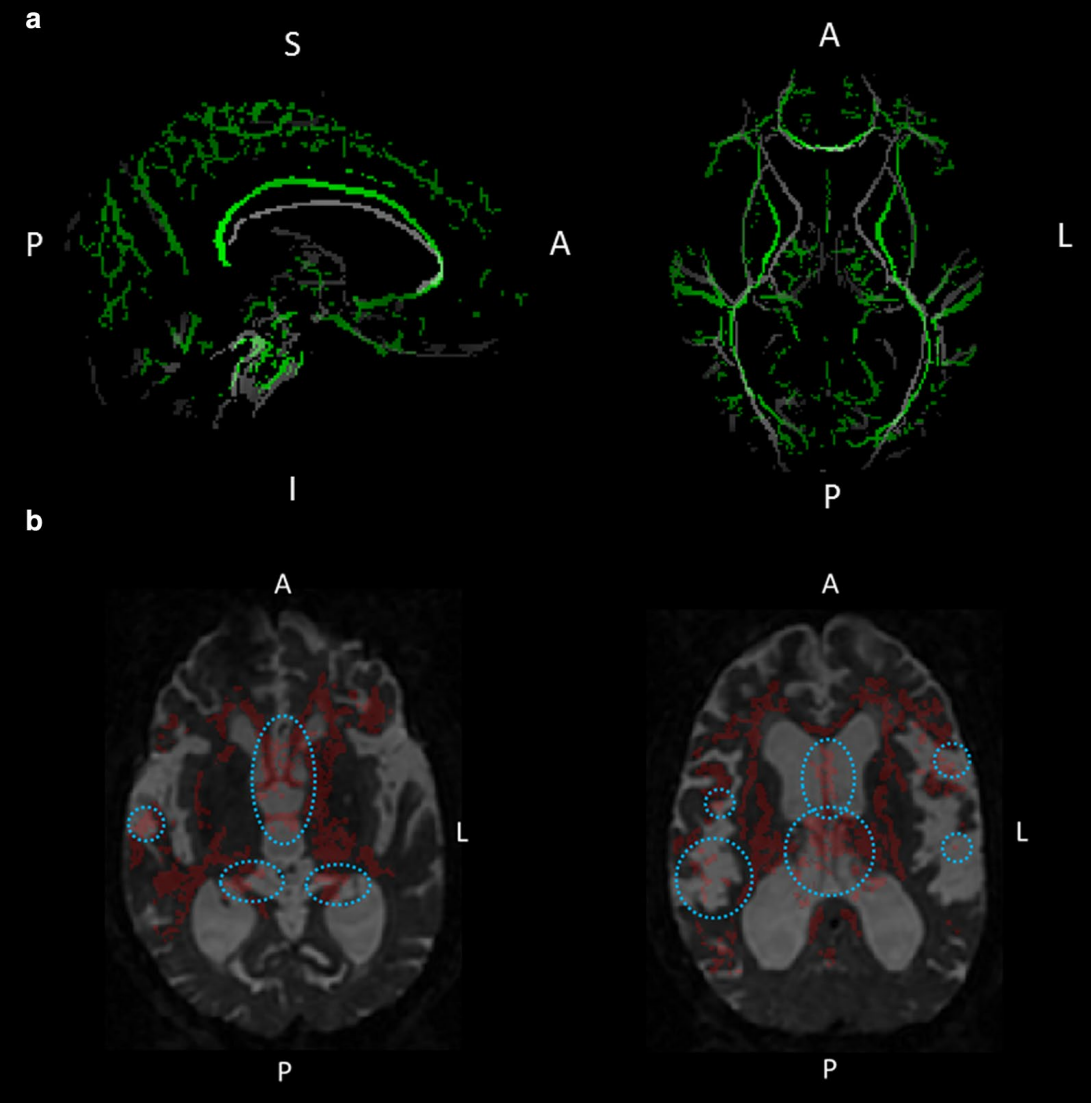
Comparison of skeletons and mis-registrations observed in TBSS analysis. **a** Demonstrates the mean FA skeleton (*green*) derived from the study (the comparison between the patients with INPH and the healthy control subjects) and the FMRIB58 FA standard space skeleton (*grey*). The periventricular tracks in the mean FA skeleton (the internal capsules and the corpus callosum in particular) are out of position. **b** Shows a sample of mis-registration that occurred in the comparison of MD maps between the patients with INPH and the healthy control subjects. The back-projected voxels (*red*) in the columns, bodies, and crura of the bilateral fornixes were positioned on each side of the lateral ventricle, and those in a peri-Sylvian portion of the SWM of bilateral frontal operculums were on each side of the Sylvian fissure. *A* anterior, *I* inferior, *L* left, *P* posterior, *S* superior

The locations of the clusters were determined first by using the Harvard-Oxford Subcortical Structural Atlas and JHU ICBM-DTI-81 White-Matter Labels (implemented in FSLView, http://www.cma.mgh.harvard.edu/fsl_atlas.html). However, there was insufficient accuracy for warping from the MNI space (Fig. 1a), such that two neurologists (S.K. and E.M.) eventually identified the locations of the clusters by consulting with each other and using the mean colour-coded FA skeleton map (directionally encoded) and the mean normalized b0 image in reference to the MRI atlas of Human White Matter made by Mori et al. [28].

To confirm whether the results of TBSS analyses were based on accurate registrations, the back-projection in TBSS was performed. The back-projection can investigate where one or more voxels of the mean FA skeleton originally came from in each subject’s FA map. Two neurologists (S.K. and E.M.) examined the clusters independently to determine whether the back-projected TBSS results were positioned on tract-centre points and were anatomically the same among the patients with INPH and the HC subjects. If both neurologists judged that some clusters in the back-projected TBSS results were positioned on the outside of tract-centre points or were different among the patients with INPH, these clusters were excluded from the data set for significant regions. In addition, anatomical ROI analyses of the regions in which FA or MD were significantly altered after shunt placement were performed using a paired two-tailed Student’s t test to validate the results of the TBSS analyses. Statistical significance was defined for p values <0.05 (using Bonferroni correction).

## Results

### Clinical assessments, ventricular size, and hemispheric DTI measures

The results of the baseline demographics and the iNPHGS, TUG, MMSE, FAB, left VV/ICV ratio, and left hemispheric FA and MD for the patients with INPH are summarized in Table 1. There were no significant differences between the SR and SNR groups, except that the number of females in the SNR group was significantly smaller than that in the SR group. The left VV/ICV ratio in the HC group (mean ratio: 1.511 ± 0.520) was significantly smaller than those in the SR (p < 0.001) and SNR (p < 0.001) groups. The left hemispheric FA in the HC group (mean FA: 0.4105 ± 0.014) was significantly larger than those in the SR (p = 0.036) and SNR (p = 0.005) groups. The left hemispheric MD (mean MD: 0.7460 ± 0.031) in the HC group was significantly smaller than those in the SR (p < 0.001) and SNR (p = 0.004) groups (data not shown). There were no significant differences in the baseline data or in the post-operative changes between the VP shunt and the LP shunt groups in the SR group (Additional file 1: Table S1).

**Fig. 2:**
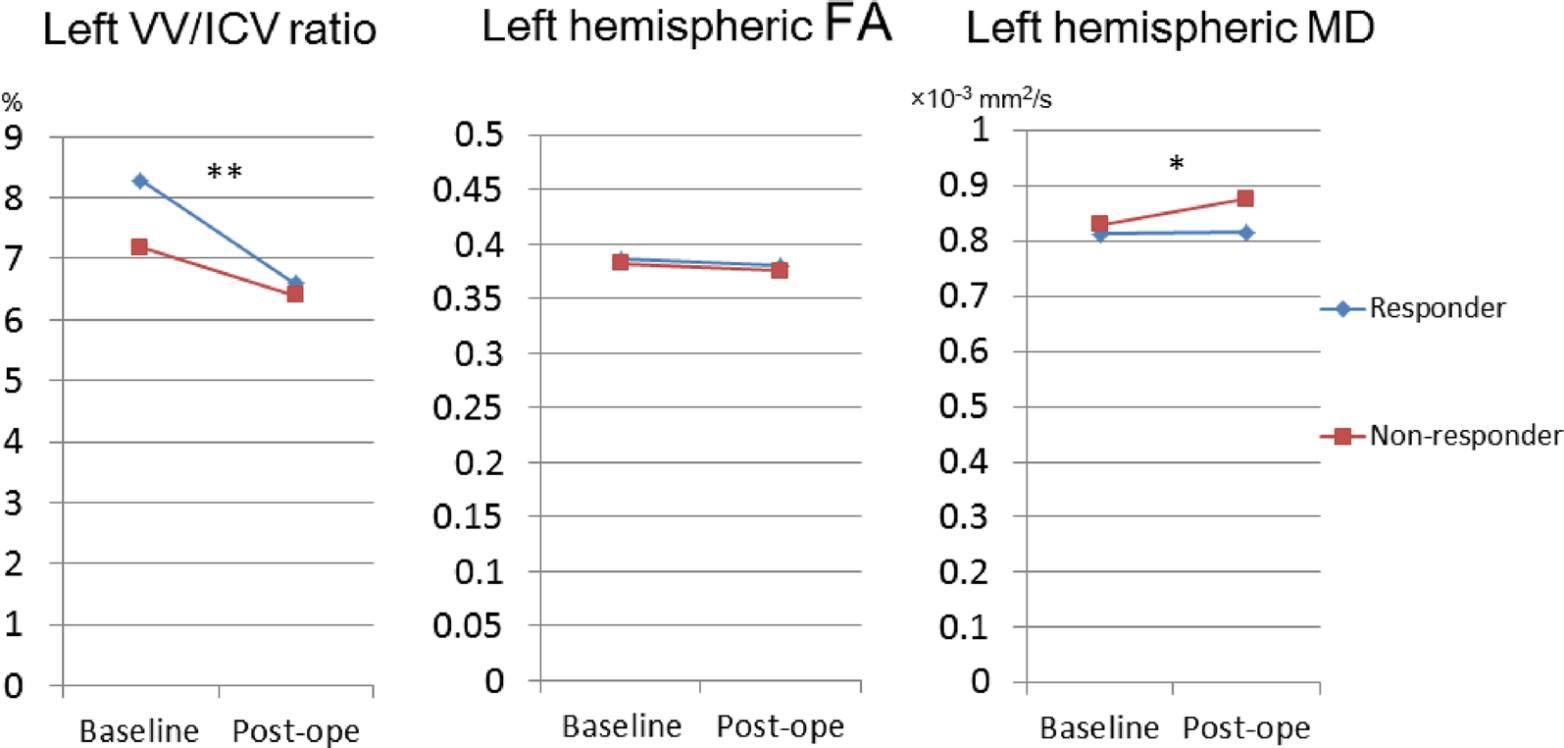
Changes in left VV/ICV ratio, hemispheric FA, and hemispheric MD after shunt placement. Significance of interaction between shunt effectiveness for clinical improvement and for each index is denoted as follows: *p < 0.05; **p < 0.001

The changes after shunt surgery in clinical and DTI measures in the patients with INPH are shown in Table 2A (SR group) and 2B (SNR group). The total and sub-scores of the iNPHGS, the total scores of the MMSE and the FAB, and the TUG complete time and number of steps in the SR group were significantly improved 1 year after shunt placement compared with those at baseline, whereas those in the SNR group were largely unchanged. The left VV/ICV ratios measured at 1 year after shunt placement were significantly smaller than those measured at baseline in both the SR and SNR groups. On the other hand, the left hemispheric FAs and MDs were not significantly changed between baseline and 1 year after shunt placement in either group. Repeated measure of ANOVA of the left VV/ICV ratio revealed a significant main effect of shunt surgery for the decrease in ventricular size [F(1, 26) = 45.11, p < 0.001] and a significant interaction between shunt effectiveness for clinical improvement and decrease in ventricular size [F(1, 26) = 5.92, p = 0.022] (Fig. 2). Although there was no significant interaction between shunt effectiveness for clinical improvement and change in the left hemispheric FA, there was a significant interaction between shunt effectiveness for clinical improvement and change in left hemispheric MD [F(1, 26) = 6.01, p = 0.021] (Fig. 2).

**Table 2:** Changes in clinical and MRI variables after shunt placement

Variables	Baseline	Post-operative	df	p value^a^
A. Shunt responders (SR, n = 20)				
TUG, mean (SD)				
Time to complete (s)	14.7 (4.1)	11.2 (4.3)	19	<0.001
Number of steps	21.9 (7.0)	18.1 (7.2)	19	0.001
iNPHGS, median (range)				
Gait disturbance	2.0 (2–3)	1.0 (0–3)		<0.001
Cognitive disturbance	3.0 (0–3)	2.0 (0–3)		0.001
Urinary disturbance	1.5 (0–3)	0.0 (0–2)		0.004
Total	6.5 (2–9)	3.0 (1–7)		<0.001
MMSE, mean (SD)	21.5 (4.6)	24.0 (4.3)	19	0.008
FAB, mean (SD)	10.7 (2.3)	12.9 (3.1)	19	0.002
Left VV/ICV ratio (%), mean (SD)	8.29 (1.99)	6.59 (2.03)	19	<0.001
Left hemispheric FA, mean (SD)	0.386 (0.022)	0.382 (0.026)	19	NS
Left hemispheric MD (×10^−3^ mm^2^/s), mean (SD)	0.813 (0.036)	0.816 (0.035)	19	NS
Mean FA of the left corona radiata, mean (SD)	0.624 (0.041)	0.592 (0.048)	19	<0.001
B. Shunt non-responders (SNR, n = 8)				
TUG, mean (SD)				
Time to complete (s)	15.6 (3.1)	14.2 (3.5)	7	NS
Number of steps	22.6 (4.0)	20.5 (5.0)	7	NS
iNPHGS, median (range)				
Gait disturbance	2.0 (1–4)	2.0 (1–4)		NS
Cognitive disturbance	2.5 (1–3)	2.0 (1–3)		NS
Urinary disturbance	2.0 (0–4)	2.0 (0–4)		NS
Total	7.0 (4–9)	7.0 (4–9)		NS
MMSE, mean (SD)	22.3 (5.0)	22.3 (5.0)	7	NS
FAB, mean (SD)	11.0 (3.8)	11.0 (3.7)	7	NS
Left VV/ICV ratio (%), mean (SD)	7.19 (1.99)	6.40 (1.79)	7	0.005
Left Hemispheric FA, mean (SD)	0.380 (0.024)	0.375 (0.030)	7	NS
Left Hemispheric MD (×10^−3^ mm^2^/s), mean (SD)	0.829 (0.068)	0.876 (0.099)	7	NS
Mean FA of the left corona radiata, mean (SD)	0.612 (0.043)	0.607 (0.044)	7	NS

*DTI* diffusion tensor imaging, *INPH* idiopathic normal pressure hydrocephalus, *iNPHGS* idiopathic normal pressure hydrocephalus grading scale, *TUG* Timed “Up and GO” test, *MMSE* Mini-Mental State Examination, *FAB* frontal assessment battery, *Left VV/ICV ratio* the volume of the left lateral ventricle + the cerebral aqueduct + the left side region of the third and fourth ventricles/the left half volume of the intracranial space, *FA* fractional anisotropy, *MD* mean diffusivity, *SD* standard deviation, *df* degrees of freedom, *NS* not significant

^a^Paired Student’s t test was used except for iNPHGS (Wilcoxon signed rank test)

### TBSS analyses

**Fig. 3:**
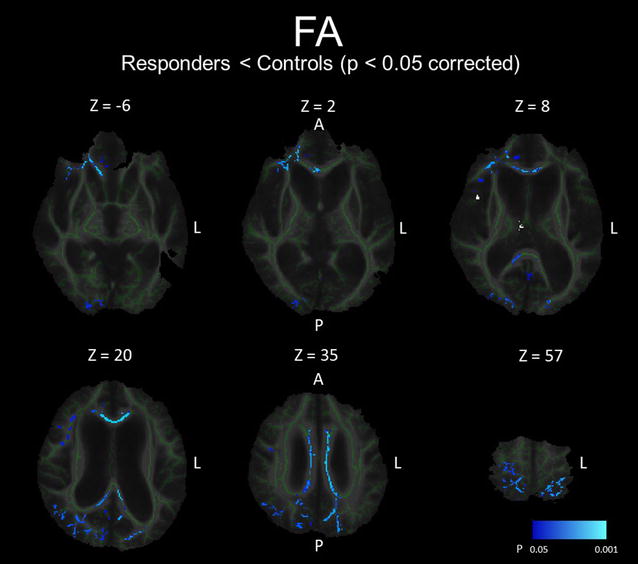
White matter regions in which FA values were significantly lower in shunt-responsive INPH. The areas with significantly lower FA values are demonstrated by colours ranging from *blue* to *light-blue* in the shunt-responsive INPH patients compared to control subjects (p < 0.05, corrected for multiple comparisons by using TFCE). The mis-registered regions are shown in *white*. *A* anterior, *L* left, *P* posterior

The comparison between FA maps for the SR group at baseline and the HC group and the regional peaks for significant clusters (t value >2.0) are demonstrated in Fig. 3 and Table 3A. The FA values of the SR group were significantly lower in the middle and posterior parts of the cingulum, genu, body, and splenium of the corpus callosum, the crus of the right fornix, and the subcortical white matter (SWM) of the right frontal operculum, right precuneus, bilateral superior parietal lobules, right occipital cortex, right primary motor cortex, and left primary motor cortex. However, as the result of the back-projection, the back-projected voxels in a peri-Sylvian portion of the SWM of the right frontal operculum were positioned on the right Sylvian fissure (incidence: HC 4/10; SR 19/20; SNR 4/10), and those in the crus of the right fornix were on the right lateral ventricle or right thalamus (incidence: HC 9/10; SR 20/20; SNR 8/8). Therefore, these regions were excluded from the FA data set. There were no regions in which the SR group presents significantly higher FA values than the HC group. In addition, there were no regions in which the FA values were significantly different between the SNR group and the HC group and between the SR group and SNR group (data not shown).

**Table 3:** Regions of the peaks for significant clusters in TBSS analyses

	Hemisphere	MNI coordinate (x, y, z)	T value^a^	Cluster size
A. FA for shunt responders (SR) at baseline compared to healthy controls (HC)				
Region				
Middle part of cingulum	Right	5, −12, 35	6.88	7210
Middle part of corpus callosum	Left	−10, −25, 38	5.56	
SWM of superior parietal lobule	Left	−22, −51, 56	5.33	
Posterior part of corpus callosum	Left	−10, −43, 33	5.31	
Posterior part of corpus callosum	Right	12, −48, 20	6.29	713
Posterior part of cingulum	Right	10, −39, 37	4.63	
Splenium of corpus callosum	Right	10, −46, 13	4.58	
SWM of precuneus	Right	9, −56, 27	5.63	113
SWM of superior parietal lobule	Right	15, −47, 52	5.55	4177
SWM of primary motor cortex	Right	23, −27, 57	4.99	
SWM of cuneus	Right	16, −74, 33	4.57	
SWM of frontal operculum	Right	43, 4, 18	4.66	387
SWM of frontal operculum	Right	42, 20, 16	4.47	358
Excluded regions				
SWM of frontal operculum	Right	41, 22, 6	7.01	39
Perithalamic white matter	Right	6, −14, 7	5.68	8
B. MD for shunt responders (SR) at baseline compared to healthy controls (HC)				
Region				
SWM of primary motor cortex	Left	−30, −14, 53	3.72	118
SWM of superior parietal lobule	Left	−13, −59, 49	3.48	119
SWM of primary motor cortex	Right	13, −20, 56	3.47	117
SWM of precuneus	Right	10, −53, 24	3.40	115
SWM of orbitofrontal cortex	Left	−26, 21, −18	3.37	114
SWM of lateral occipital cortex	Right	38, −63, 27	3.35	111
Cingulum (cingulate gyrus)	Right	7, −26, 37	3.01	106
Cerebral peduncle	Left	−10, −17, −7	2.74	108
SWM of middle temporal gyrus	Right	51, −42, 4	2.74	101
External capsule	Right	23, 20, −4	2.73	112
SWM of superior frontal gyrus	Left	−18, −1, 47	2.63	103
SWM of superior parietal lobule	Right	42, −53, 39	2.58	94
Posterior corona radiata	Right	35, −40, 36	2.43	87
Superior longitudinal fasciculus	Left	−30, 15, 30	2.38	100
Perithalamic white matter	Left	−18, −22, 9	2.32	83
SWM of inferior parietal lobule	Right	58, −28, 31	2.28	105
SWM of superior temporal gyrus	Right	51, −16, −2	2.17	98
SWM of primary sensory cortex	Left	−9, −45, 59	2.14	39
SWM of precuneus	Left	−8, −58, 42	2.10	96
Excluded regions				
Cingulum (hippocampus)	Right	26, −41, −6	14.4	120
Fornix	Right	3, −20, 20	10.6	120
Fornix	Left	0, −20, 21	10.6	
SWM of frontal operculum	Right	54, −10, 14	5.57	116
C. FA in shunt responders (SR) post-operation compared to baseline				
Corona radiata	Left	−27, −10, 15	6.93^b^	66

*DTI* diffusion tensor imaging, *INPH* idiopathic normal pressure hydrocephalus, *HC* healthy controls, *MNI* Montreal Neurological Institute, *FA* fractional anisotropy, *MD* mean diffusivity, *SR* shunt responder, *SWM* subcortical white matter

^a^The number of degrees of freedom is 28

^b^The number of degrees of freedom is 19

**Fig. 4:**
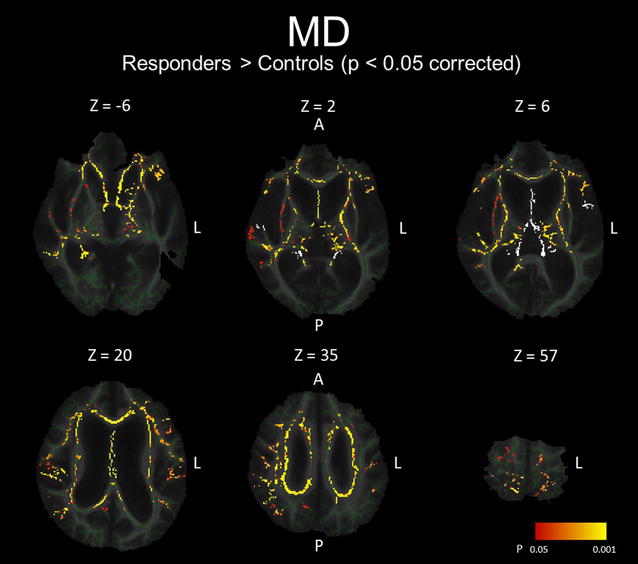
White matter regions in which MD values were significantly higher in shunt-responsive INPH. The areas with significantly higher MD values are demonstrated by colours ranging from *red* to *yellow* in the shunt-responsive INPH patients compared to control subjects (p < 0.05, corrected for multiple comparisons by using TFCE). The mis-registered regions are shown in *white*. *A* anterior, *L* left, *P* posterior

Figure 4 and Table 3B show the results of the group comparison for MD between the baseline SR and HC groups and the regional peaks for significant clusters (t value >2.0). The MD values of the SR group were significantly higher in the columns, bodies, and crura of the bilateral fornixes, the hippocampal part of the right cingulum, the genu, body, and splenium of the corpus callosum, and the subcortical white matter (SWM) of the bilateral frontal operculum, bilateral orbitofrontal cortex, bilateral primary motor and sensory cortex, posterior part of temporal cortex, bilateral superior and inferior parietal lobules, right precuneus, and right occipital cortex, as well as the right external capsule, the bilateral cerebral peduncles, and the bilateral internal capsules. However, as a result of the back-projection, the back-projected voxels in the columns, bodies, and crura of the bilateral fornixes were positioned on each side of the lateral ventricle (incidence: HC 10/10; SR 20/20; SNR 8/8), those in a peri-Sylvian portion of the SWM of bilateral frontal operculums were on each side of the Sylvian fissure [incidence (right): HC 5/10; SR 20/20; SNR 8/8, incidence (left): HC 0/10; SR 20/20; SNR 8/8], and those in the SWM of posterior part of the right temporal cortex were on the right Sylvian fissure (incidence: HC 0/10; SR 20/20; SNR 8/8). Therefore, these regions were excluded from the MD data set (Fig. 1b). There were no regions in which the SR group presented a significantly lower MD than the HC group. Furthermore, there were no regions in which the MD values were significantly different between the SNR group and the HC group and between the SR group and SNR group (data not shown).

**Fig. 5:**
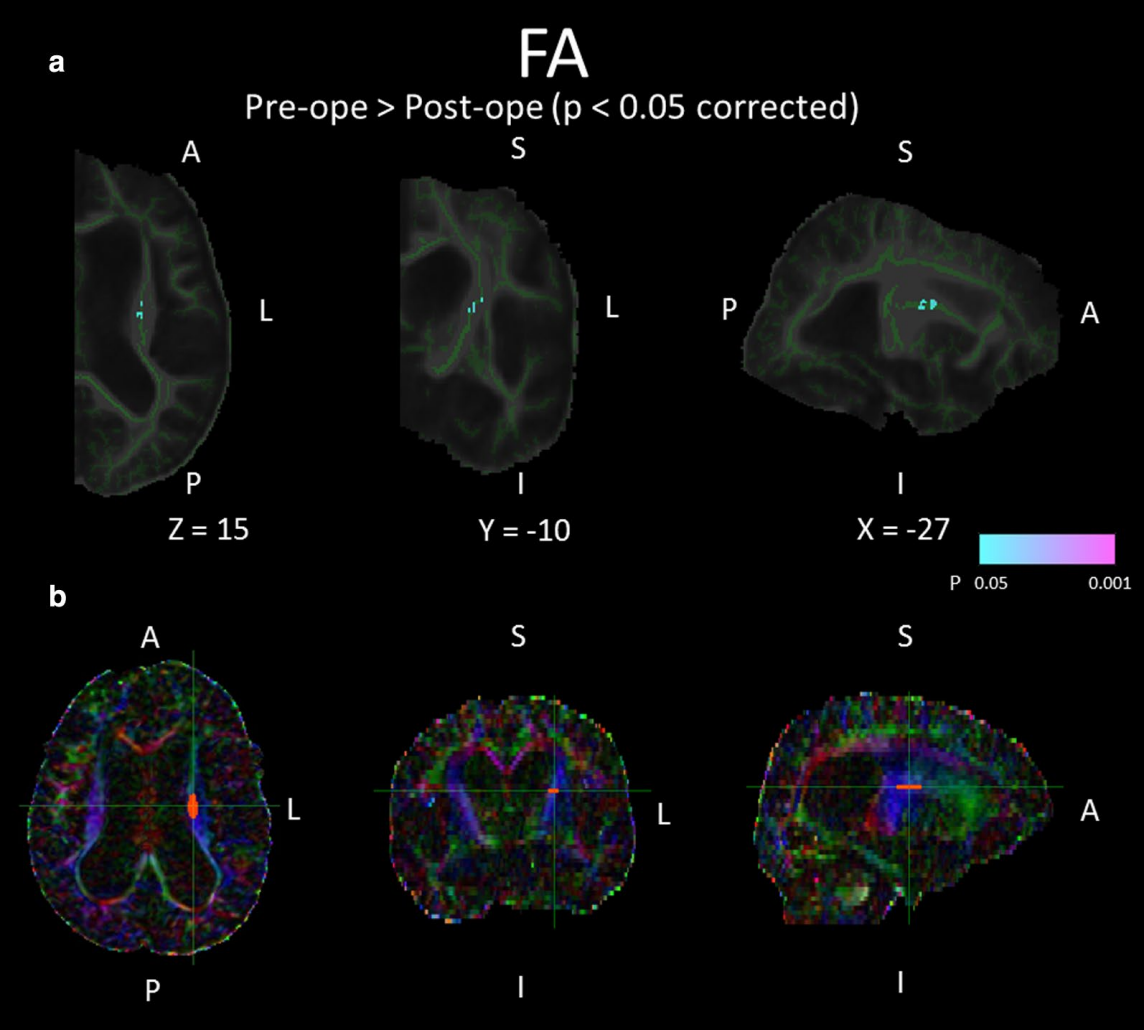
White matter regions in which FA values significantly changed after shunt placement. **a** Demonstrates the areas with significantly decreased FA values after shunt placement in the shunt-responsive INPH patients using colours ranging from* light-blue* to* pink* (p < 0.05, corrected for multiple comparisons by using TFCE). **b** Shows the position of the anatomical ROI. On colour-coded FA map, *red*, *green*, and *blue* represent the direction of fibres (*red*, *right*-*left*; *green*, anterior-posterior; *blue*, superior-inferior). The ROI was placed on the *blue* regions in the left radiata on the axial slice that included the genu of the corpus callosum. *A* anterior, *L* left, *P* posterior

The comparison of FA maps between baseline and 1 year after shunt placement in the SR group are shown in Fig. 5a and Table 3C. The FA values were significantly decreased in the left corona radiata. In addition, the result of the back-projection revealed the precise registration of each FA map. The anatomical ROI of the left corona radiata of each SR or SNR patient was placed using colour-coded FA map and referring to the result of the TBSS analysis (Fig. 5b). The anatomical ROI analysis showed that the mean FA value of the regions in the left corona radiata was significantly decreased after shunt placement only in the SR group (Table 2A). There were no other regions in which the FA values were significantly increased after shunt placement in the SR group. In the comparison of the FA map between baseline and 1 year after shunt placement in the SNR group, there were no regions in which the FA values were significantly altered. In the comparison of MD maps between baseline and 1 year after shunt placement, there were no regions in which the MD values were significantly altered in either the SR or the SNR groups.

## Discussion

In the present study, we investigated the differences between shunt responders and non-responders after shunt placement by applying the TBSS including back-projection and hemispheric DTI analyses to elucidate brain white matter changes in INPH patients with shunt placement. We showed that FA values in the corona radiata were decreased by shunt placement in patients that had a good response to shunt treatment. In the back-projection analysis of the TBSS, mis-registration was observed in the periventricular or intraventricular structures such as the fornix, hippocampal part of the cingulum, perithalamic area, and peri-Sylvian SWM. These regions appear to be strongly associated with the typical deformation of the brain in patients with INPH [30]. The back-projected voxels in the fornix, cingulum, and perithalamic area were positioned on each side of lateral ventricle. These findings of mis-registration were also reported in the previous INPH study applying the TBSS analysis [15]. Hattori et al. speculated that the fornix might be displaced by the elevated and stretched corpus callosum because the fornix is attached to the posterior part of the body and splenium of the corpus callosum [15]. Not only the ventricles but also the Sylvian fissures are commonly dilated in INPH, which might cause inhibition of lateral expansion of the lateral ventricles represented by the narrowing of the callosal angle [31, 32]. The dilation of the Sylvian fissures might be one cause of the elevation of the corpus callosum.

On the other hand, the results of back-projection revealed the precise registration of the corpus callosum, the cerebral peduncles, the internal capsule, the corona radiata, and the SWM except for the frontal and temporal operculum. The DTI measures of the corpus callosum and the CST using the TBSS were validated by anatomical ROI analysis in several previous studies [10, 15]. The results of the current study suggested that the TBSS is a useful tool for detecting white matter abnormalities in these regions.

FA values in the corpus callosum and in the SWM of the convexity and occipital cortex were significantly lower in SR than in HC, whereas MD values in the periventricular and peri-Sylvian white matter were significantly higher. These findings were frequently reported in the previous DTI studies [10, 15]. The white matter regions in which FA values were decreased after shunt placement were distributed in the left corona radiata. These regions were located between the lateral ventricle and the Sylvian fissure, and therefore may have been severely compressed. Recent studies reported that the FA values of the CST near the lateral ventricles in INPH were higher compared with those in healthy controls, although this result was not confirmed in the present study [10, 11]. Hattori et al. detected the increase of axial eigenvalues and unaltered radial eigenvalues in the CST and suggested that mechanical pressure from ventricular dilatation could enhance directional water diffusivity parallel to the axon in the CST in INPH [11]. In addition, we speculated that dilatation of the Sylvian fissures could play a role of counterfort against mechanical pressure from the ventricular dilatation in INPH. It is considered that the release of compression to the corona radiata including the CST and projection fibre to the medial frontal cortex, which is thought to play a role in planning or programming voluntary movements including gait, is associated with clinical improvement after shunt placement [9, 33, 34].

Although the left hemispheric FA and MD of the INPH patients were not significantly changed after shunt placement, it was identified that left hemispheric MDs of the SNR patients increased after shunt placement when compared to those of the SR patients. In addition, it was revealed that the ventricles of the SNR patients decreased to a lesser extent after shunt placement compared to those of the SR patients which started from a higher preshunt ratio. One of the possible reasons is insufficiency of pressure adjustments in clinical practice. In the study, all the patients in the SNR group fulfilled the clinical criteria of the Japanese Clinical Guidelines for INPH. In previous cohort studies in Japan using the same criteria, the efficiency of the shunt operation (both VP and LP shunts) was 80%. Because the efficiency in the current study was 71% (20/28), there was no apparent evidence that the pressure adjustments were insufficient [29, 30]. Another possible reason is that these findings reflected progression of brain atrophy in SNR. Poor contraction of ventricles and an expansion of the interstitial oedema represented by an increase in the MD value might be related to a decline in compliance of the brain, which is associated with co-morbidity with other central nervous system diseases. Hamilton et al. reported that Alzheimer disease pathology observed in cortical biopsies obtained during shunt insertion was associated with worse baseline cognitive performance and diminished postoperative improvement in patients with hydrocephalus [35]. We excluded the patients with vascular brain disease from the study by neuroradiological criteria. However, the severity of ischaemic brain injuries might be relevant to shunt responsiveness because micropathological findings of ischaemic demyelination and infarction have been observed in the brains of patients with normal pressure hydrocephalus [5]. In the future, we need to investigate whether the pre-operative hemispheric FA and MD can predict the effectiveness of shunt surgery in a large-scale cohort study, which will lead us to better outcomes and further discussions about neuropathological changes of the cerebral white matter in INPH.

In our previous study, we reported that the hemispheric FA in INPH was lower than that in disease controls (Alzheimer’s disease and idiopathic Parkinson’s disease), was correlated with severity of gait and cognitive disturbances, and was not correlated with degree of ventricular dilation [9]. According these findings, it was suggested that the hemispheric FA might be altered depending on improvement of gait and cognitive disturbances. However, the results in the present study were inconsistent with our expectation. Although the hemispheric FA in INPH might strongly reflect the severity of irreversible white matter involvement, further studies are needed to clarify this issue.

There were several limitations in the current study: one is that we could not detect a change in white matter in the right brain after shunt placement because of shunt valve artefacts. It is possible that the change in white matter after shunt placement is different between the right and left brains, although the brain ventricular system consists of a semi-enclosed cavity. One solution to this problem may be to analyse DTI of only subjects who underwent lumboperitoneal (LP) shunt surgery. In this study, there were no significant differences in the changes of the clinical and hemispheric DTI data after shunt placement between the LP and VP shunt groups. A recent study revealed the benefit of LP shunt surgery for patients with INPH [29]. However, there were insufficient patient numbers to conclude whether the effectiveness of an LP shunt is equivalent to that of a VP shunt. In addition, regional statistical comparisons of the change in the DTI parameters after shunt placement between the LP and VP shunt groups could not be performed because of insufficient sample sizes. Further larger-scale studies are needed to confirm whether there are significant differences in clinical effectiveness and of regional changes in DTI parameters between the LP and VP shunts.

Another limitation is that the mean age of the HC subjects was significantly lower than that of the INPH patients. Previous studies suggested that the FA values tend to decline and the MD value tend to elevate with ageing [26]. Although we applied age as nuisance variable in the comparison analysis, the results of the DTI analyses might overestimate the cerebral white matter change in INPH. The other limitation is the small sample size of the SNR group. There is some possibility that we could not detect the difference in white matter alterations between the SR and SNR groups and between the SNR and HC groups. Moreover, another limitation is that we applied the non-isotropic voxels and only 13 gradient directions in the DTI scan. The FA values measured using non-isotropic voxels tend to result in underestimation in regions with crossing fibres such as the centrum semiovale (crossing interhemispheric, projection, and association fibres) [36]. In addition, the analysis of high resolution DTI using non-isotropic voxels may not be sufficiently precise because the signal-to noise ratio is prone to decline with a decrease in the voxel size [37]. Melhem et al. suggested that more than 20 gradient directions were needed to obtain highly reproducible FA values [37]. These problems might be solved by using iso-tropic voxels or a many gradient directions scan. However, this seems to be difficult in clinical settings.

## Conclusions

Our study demonstrated that fractional anisotropy in the corona radiata was decreased by shunt placement. The regions were distributed between the enlarged lateral ventricles and Sylvian fissures. This finding was observed only in shunt-responsive INPH patients and might reflect the plasticity of the brain for mechanical pressure from the cerebrospinal fluid system.

## Additional file

**Additional file 1: Table S1.** VP shunt versus LP shunt (shunt responders).

## Abbreviations

CSF: cerebrospinal fluid
CST: corticospinal tract
DTI: diffusion tensor imaging
DWI: diffusion-weighted image
FA: fractional anisotropy
FAB: frontal assessment battery
FLAIR: fluid attenuated inversion recovery
HC: healthy control
INPH: idiopathic normal pressure hydrocephalus
iNPHGS: idiopathic Normal Pressure Hydrocephalus Grading Scale
LP: lumboperitoneal
MD: mean diffusivity
MMSE: Mini-Mental State Examination
MNI: Montreal Neurological Institute
ROI: region of interest
SNR: shunt non-responder
SR: shunt responder
SWM: subcortical white matter
TBSS: tract-based spatial statistics
TFCE: threshold-free cluster enhancement
3D-SPGR: three-dimensional spoiled gradient echo
TUG: Timed Up and Go Test
VP: ventriculoperitoneal
VV/ICV: volume of ventricles/intracranial volume
