# Supplementary material for: A change in brain white matter after shunt surgery in idiopathic normal pressure hydrocephalus: a tract-based spatial statistics study
Source: Fluids Barriers CNS. 2017 Jan 30;14:1. doi: 10.1186/s12987-016-0048-8 (PMC5278569; doi:10.1186/s12987-016-0048-8)
Supplement: Supplementary file 1 — Additional file 1: Table S1. VP shunt versus LP shunt (shunt responders). [file 12987_2016_48_MOESM1_ESM.docx]

Supplementary table 1. VP shunt versus LP shunt (shunt responders).

| Variables | VP shunt  (n = 13) | LP shunt  (n = 7) | df | P-value^a^ |
| --- | --- | --- | --- | --- |
| Baseline data  Age in years, mean (SD)  Sex, female/male  Education years, mean (SD)  TUG, mean (SD)  Time to complete (sec)  Number of steps  iNPHGS, median (range)  Gait disturbance  Cognitive disturbance  Urinary disturbance  Total  MMSE, mean (SD)  FAB, mean (SD)  Left VV/ICV ratio (%), mean (SD)  Left Hemispheric FA, mean (SD)  Left Hemispheric MD (×10^-3^ mm^2^/s) , mean (SD)  Post-operative changes  TUG, mean (SD)  Time to complete (sec)  Number of steps  iNPHGS, median (range)  Gait disturbance  Cognitive disturbance  Urinary disturbance  Total  MMSE, mean (SD)  FAB, mean (SD)  Left VV/ICV ratio (%), mean (SD)  Left Hemispheric FA, mean (SD)  Left Hemispheric MD (×10^-3^ mm^2^/s), mean (SD) | 75.7 (5.4)  9/4  10.0 (2.4)  14.4 (4.1)  21.7 (7.4)  3.0 (2-3)  3.0 (1-3)  1.0 (0-3)  7.0 (4-9)  21.0 (5.1)  10.6 (2.7)  8.30 (2.07)  0.386 (0.024)  0.812 (0.039)  -2.9 (1.0)  -2.8 (3.3)  -1.0 (-2-0)  -1.0 (-2-0)  -1.0 (-3-0)  -3.0 (-5--1)  2.5 (4.0)  2.6 (2.9)  -1.87 (1.08)  -0.004 (0.017)  0.000 (0.016) | 75.0 (5.2)  3/4  11.0 (3.7)  15.2 (4.5)  22.2 (7.0)  2.0 (2-3)  2.0 (0-3)  2.0 (0-3)  5.0 (2-8)  22.4 (7.0)  10.7 (1.4)  8.28 (1.99)  0.385 (0.033)  0.813 (0.033)  -4.5 (3.2)  -5.5 (4.4)  -1.0 (-1--1)  0.0 (-2-0)  0.0 (-2-1)  -2.0 (-5--1)  2.7 (3.4)  2.1 (2.7)  -1.39 (0.74)  -0.003 (0.013)  0.008 (0.025) | 18  1  18  18  18  18  18  18  18  18  18  18  18  18  18  18  18 | NS  NS  NS  NS  NS  NS  NS  MS  NS  NS  NS  NS  NS  NS  NS  NS  NS  NS  NS  NS  NS  NS  NS  NS  NS |

INPH = idiopathic normal pressure hydrocephalus; iNPHGS = idiopathic normal pressure hydrocephalus grading scale; VP = ventriculoperitoneal; LP = lumboperitoneal; TUG = Timed “Up and GO” test; MMSE = Mini-Mental State Examination; FAB = Frontal Assessment Battery; Left VV/ICV ratio = the volume of the left lateral ventricle + the cerebral aqueduct + the left side region of the third and fourth ventricles / the left half volume of the intracranial space; FA = fractional anisotropy; MD = mean diffusivity; NS = not significant; SD = standard deviation.

^a^Student’s t test was used except for sex (Chi-square test ) and iNPHGS (Wilcoxon signed rank test).
